# Emergency Response and Risk Communication Effects of Local Media during COVID-19 Pandemic in China: A Study Based on a Social Media Network

**DOI:** 10.3390/ijerph182010942

**Published:** 2021-10-18

**Authors:** Lei Jiang, Yujia Huang, Haonan Cheng, Ting Zhang, Lei Huang

**Affiliations:** State Key Laboratory of Pollution Control and Resource Reuse, School of the Environment, Nanjing University, Nanjing 210023, China; jianglei@smail.nju.edu.cn (L.J.); yujiahuang123@gmail.com (Y.H.); chnchenghaonan@163.com (H.C.); zhangting@nju.edu.cn (T.Z.)

**Keywords:** emergency response, risk communication, COVID-19, public participation, social media

## Abstract

As the country where the COVID-19 was first reported and initially broke out, China has controlled the spread of the pandemic well. The pandemic prevention process included emergency response and risk communication, both of which could notably increase public participation, people’s anxiety has been alleviated, their confidence in the government has been enhanced, and the implementation of prevention and control measures has been understood. This study selected 157,283 articles published by 447 accounts across 326 cities in February 2020 from WeChat, the largest social media application in China, to systematically compare the spatial distributions in the effectiveness of emergency responses and risk communication. The results showed that there were significant regional differences in the effectiveness of emergency response and risk communication during the pandemic period in China. The effectiveness of emergency response and risk communication are related to the exposure risk to the COVID-19, the level of economy, culture, and education of the region, the type of accounts and articles, and the ranking of the articles in posts. The timeliness and distribution types of articles should take into account the psychological changes in communication recipients to avoid the dissemination of homogenized information to the masses and the resulting information receiving fatigue period.

## 1. Introduction

The novel coronavirus, COVID-19, which first broke out in Wuhan, China, quickly spread across the world. The COVID-19 pandemic has a profound impact on economic development, industrial manufacturing, and public wellbeing [1,2]. By January 2021, the accumulative detected COVID-19 infections had exceeded 100 million worldwide. The prevention and control of the COVID-19 is a governance issue, which requires the coordination of central and local governments, various social organizations, and the public [3]. Compared to medical intervention, non-medical interventions, such as the use of face masks, social distancing, and environmental disinfection, are more effective in the early stages of pandemic control [4,5,6]. In order to restore normal life as soon as possible, non-pharmacological interventions based on public behaviors are the key to prevent and control pandemics until vaccines are widely used and herd immunity is achieved [7,8,9]. Due to political, economic, cultural, historical, and traditional reasons, there are significant differences in the emergency response measures among countries [3,10]. China has taken comprehensive measures to control the pandemic through population mobility management, health education, personal protection, and environmental disinfection [11,12]. Instead of an immediate nationwide lockdown, the EU adopted information sharing, protection resource management, and economic measures in a united response to the pandemic [13,14]. In the fight against the COVID-19, different countries have developed corresponding pandemic control measures by promoting public participation with emergency response and risk communication.

Emergency response and risk communication are important in terms of pandemic management [15]. Emergency response refers to the speed and the effectiveness of the response of the public sector after the occurrence of an emergency. Emergency response includes emergency management measures and information dissemination, etc. [16]. Risk communication is the communication between the public sector and the public on issues concerning emergency prevention methods, response measures, protection matters, and post-event arrangements. Risk communication is employed to achieve consensus and to promote the efficiency of emergency handling [17]. Therefore, the emerging public health crisis that was related to COVID-19 is also a challenge for risk management and risk communication [18]. Effective emergency response and risk communication are important measures to obtain crisis-related knowledge, overcome public tension, and enhance confidence so as to improve public participation in emergencies [19,20,21].

Social media plays an important role in promoting public participation and is a favored tool in terms of emergency response and risk communication [21,22]. Existing studies have verified the ability of social media in promoting public participation and developing health behavior management, risk perception, and the protection of priority populations [23,24,25]. Meanwhile, the accuracy of and the emotion in social media information have direct impacts on the communication effect, which may even lead to adverse situations [26,27,28]. During the COVID-19 pandemic, information from social media has been important for the public to understand and evaluate the pandemic; inevitably, misinformation from some media sources also aggravated public anxiety and reduced the effectiveness of other risk management measures [29,30,31]. The public’s ability to distinguish misinformation is limited, leading to the widespread of such misinformation [32]. To achieve effective risk communication amid complex information, the government and non-governmental organizations need to apply proactive strategies regarding social media [33,34,35,36,37]. Only high-frequency, effective, and reliable information can fundamentally enhance the public’s confidence in the government and alleviate their anxiety levels [38,39,40,41]. Additionally, such information encourage the public to cooperate with the government’s actions.

In the course of China’s fight against the COVID-19, the pandemic was quickly controlled under the strong control of the Chinese government [42]. Emergency response and risk communication were important for the control of COVID-19 in China, and the use of social media played a prominent role in this process [33,43,44]. The timely release of domestic and global pandemic situations, practical health guidelines, and coping strategies developed by the government can greatly shape the confidence and ability of the whole society in response to the pandemic [11,33,45,46,47]. This study aims to analyze the regional differences in China by reviewing the development of emergency response and risk communication in different parts of China, so as to provide a reference for future crisis management.

## 2. Materials and Methods

### 2.1. Study Object

WeChat, a free multiple-purpose app developed by Tencent, Inc., integrates instant messaging, payment, and social public platforms. By 2021, WeChat had 1.09 billion daily active users, making it the most popular social medium in China. In the battle against the COVID-19 in China, WeChat played an important role in emergency response and risk communication due to its extensive user coverage and quick ability to spread speed news [48]. WeChat official accounts are a feature of WeChat that allows institutions and individuals to create accounts to push articles. In this research, we investigated the articles posted by WeChat official accounts to study the effects of the articles posted by different types of media accounts on WeChat regarding pandemic emergency response and risk communication. We comprehensively considered the credibility and the audience coverage of these accounts as well as the two-level pandemic management system of the local provinces and municipalities in China [49,50]. The account type was classified according to the unit to which the WeChat account belongs. Specifically, government accounts, news media accounts, and provincial-level health commission accounts were chosen. Government accounts and news media accounts were selected at the municipal level. However, some small cities did not have both a government account and a news account at the same time. Therefore, for all of the small cities, we only chose one account per city for this research. Additionally, in these small cities, government accounts were preferred, and news media accounts would be used when government accounts were unavailable.

### 2.2. Data Sources

The content and corresponding data released by WeChat official accounts were obtained from the WeChat public platform through the WeChat official account content service company. The daily number of newly confirmed cases of the COVID-19 was obtained from the official website of the Health Commission. The education level of the enrolled cities was represented using the China Education Index [51], while the culture level was represented using the China City Culture Development Index [52]. Other socioeconomic data were obtained from local Statistical Yearbooks and the official websites of local governments.

### 2.3. Measures of Emergency Response and Risk Communication

Any given public WeChat account can post one to several articles to their subscribers at any time. Subscribers can read article(s) of interest and decide whether to choose the “wow” option for each article or not. By February 2020, articles on WeChat were marked with two categories: “read” and “wow”. “Read” indicates how far an article has spread and how many people have read it. “Wow” represents whether readers like the article or not. If a reader clicks “wow”, the article is shared among the friends of the reader and can reach more potential readers [53]. It is possible that “Wow” reflects the value and degree of empathy of the article [54].

The dissemination effect of WeChat official account articles was used to reflect the effect of emergency response and risk communication based on the location of the account. The number of articles (NA) and the number of posts (NP) from WeChat official accounts were selected as a quantitative description of local emergency response in different places. As for the assessment of the effect of risk communication, the average reading quantity (ARQ) and the average wow quantity (AWQ) of articles on WeChat official accounts were used to represent the communication effect of these accounts.

### 2.4. Data Preprocessing

#### 2.4.1. Identification of Articles Related to the COVID-19

Although fighting COVID-19 was the main situation in China, not all the articles were related to the pandemic. Therefore, articles that were related to the pandemic needed to be screened out first. Keywords related to the pandemic were used as the retrieval content to retrieve the titles and contents of the articles published by official accounts so as to determine the articles related to the pandemic. The keywords were Chinese words related to the pandemic, such as the COVID-19, confirmed, asymptomatic infected, etc.

#### 2.4.2. Region Classification

To explore regional differences in COVID-19 emergency response and risk communication in China, we divided the provinces and cities into six sections based on the geographical distance commonly used in China. The six parts are North China (NC, including Beijing, Tianjin, Hebei, Shanxi, and Inner Mongolia), Northeast China (NE, including Liaoning, Jilin, and Heilongjiang), East China (EC, including Shanghai, Jiangsu, Zhejiang, Anhui, Fujian, and Shandong), Central China (MC, including Henan, Hubei, Hunan, and Jiangxi), South China (SC, including Guangdong, Hainan, and Guangxi), and West China (WC, including Chongqing, Sichuan, Guizhou, Yunnan, Shaanxi, Gansu, Qinghai, and Ningxia).

#### 2.4.3. City Classification

Chinese cities vary greatly in terms of administrative levels and sizes. The impact of such differences on pandemic prevention and control should be considered [55,56]. Therefore, cities with a high administrative level and large scale were unified as key cities, which refer to municipalities, provincial capitals, and municipalities with independent planning status under national social and economic development (MI-city). Here MI-city refers to a city that belongs to an ordinary city in terms of administration but also enjoys the status of being a provincial-level economic management authority city. The rest of the cities were analyzed as ordinary cities.

#### 2.4.4. Article Content Classification

Word frequency statistics were conducted regarding the titles of selected articles. After manually removing the words that were irrelevant to the division of the content related to the pandemic, the 100 pandemic-related words with the highest occurrence frequency were selected. The content of the articles was divided into five categories according to the content attribution of the related words. Considering that several related words of different categories may appear in one title simultaneously, we assigned priority to the five categories. The priority of the selected categories was pandemic prevention knowledge > work and living arrangements > confirmed pandemic information > pandemic news > other.

In addition, WeChat official accounts can push several articles once time, and the article headline has a more obvious display effect. Therefore, the selected articles were divided into headlined and non-headlined to analyze the difference between emergency response and risk communication effects.

#### 2.4.5. Data Classification

In the study of the influencing factors, the influencing factors were divided into five levels. Gross Domestic Product (GDP) was divided into CNY 0 to 50 billion, CNY 50 to 100 billion, CNY 100 to 500 billion, CNY 500 to 1000 billion, and over CNY 1000 billion. The permanent resident population was classified into 0 to 5 million, 5 to 10 million, 10 to 50 million, 50 to 100 million, and more than 100 million. Other factors were equally divided into 5 parts.

In order to study the differences in the effect of emergency response on risk communication, WeChat public accounts were equally divided into three groups according to the number of accounts: high, middle, and low, and then the effects of risk communication were studied. When grouping the accounts, the public accounts were divided into three groups of equal number according to NA and NP.

### 2.5. Data Analysis and Visualization

Mann–Whitney U tests and Kruskal–Wallis H tests were used to analyze the regional differences and influencing factors of emergency response and risk communication [45,57]. The analysis was conducted using SPSS 26. The diagrams were drawn using Python with PyEcharts.

## 3. Results and Discussion

### 3.1. Descriptions of Data

The data included 447 WeChat official accounts from 326 cities in 29 provinces (including autonomous regions and municipalities). The official accounts involved 87 provincial accounts and 372 municipal accounts. Municipalities were double-counted since they were both provinces and cities in China. In terms of official account types, there were 418 government accounts (including news media accounts) and 29 provincial health commission accounts. In February 2020, all of the accounts posted 157,283 articles for a total of 49,224 post times, of which 150,144 were related to the pandemic, accounting for 95.46% of the posted articles, generating 1,599,904,735 views (96.47%) and 8,433,730 wows (95.54%).

### 3.2. Emergency Response Effects

The statistical analysis of the emergency response effects across China in February 2020 are listed on Table 1.

#### 3.2.1. The Differences of Emergency Response in WeChat Accounts at Different Types

**Figure 1:**
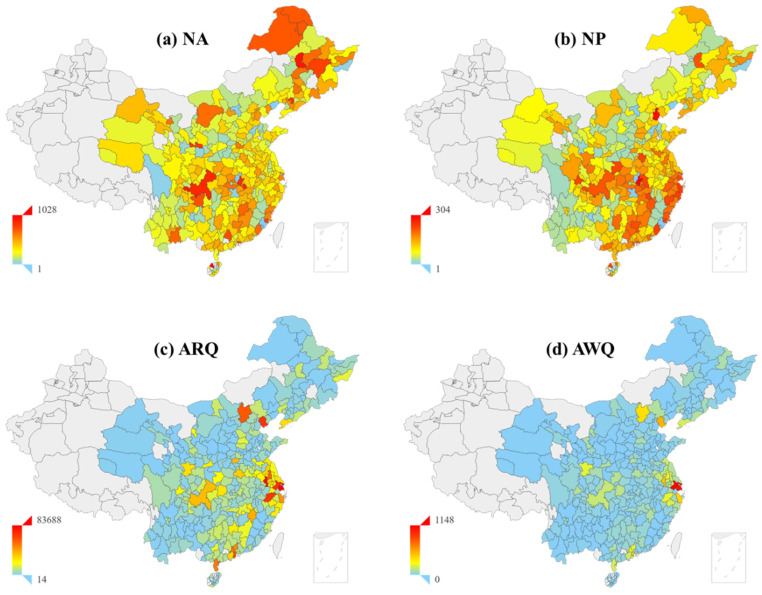
Regional distribution of emergency response and risk communication effects. Note: (**a**) The distribution of the number of articles (NA) in Chinese cities. (**b**) The distribution of the number of posts (NP) in Chinese cities. (**c**) The distribution of the average reading quantity (ARQ) of the articles in Chinese cities. (**d**) The distribution of the average wow quantity (AWQ) of articles in Chinese cities.

The regional differences in emergency response are divided in terms of provincial and city prospects. In terms of provincial media, there are obvious regional differences in NA by news media accounts (*p* = 0.017), while the distribution of the NA (*p* = 0.873) and NP (*p* = 0.761) of articles published by the government is independent of the region. Moreover, there is a near significant regional difference in the NP between the news media accounts (*p* = 0.054) and the health commission accounts (*p* = 0.064). From the city perspective, there are significant regional differences in the NA (*p* = 0.027) and NP (*p* < 0.001) of all of the urban media accounts, as shown in Figure 1a–d. EC, MC, SC, and NE were higher than NC and WC in terms of the number of articles. EC, MC, and SC were higher than NE, NC, and WC in terms of the number of posts. NE posted more articles at a lower frequency. Nevertheless, for NA and NP, there are no significant regional differences among the government accounts and news media accounts of key cities.

The regional differences in the emergency response of various media accounts to the pandemic are influenced by local development levels, the development of the pandemic, and the government’s response policies. This influence is well demonstrated on provincial WeChat accounts among different regions. The pressure to control the pandemic was first applied by the government and then on the health commission, while the news media is under the least pressure. Therefore, there are no significant regional differences in terms of the government accounts. However, the NP of the health commission accounts is about to be significant, while there are significant regional differences in the NA of news media accounts, and the NP is about to be significant. The health commission is the government’s health and wellness authority that does the actual work, and its emergency response capacity at the social media level has been reduced. From the urban point view, the emergency response not found in key cities is not differentiated by region but is different among all cities, indicating that the gap in emergency response is related to the comprehensive level of cities.

#### 3.2.2. The Differences of Emergency Response in Cities at Different Levels

**Figure 2:**
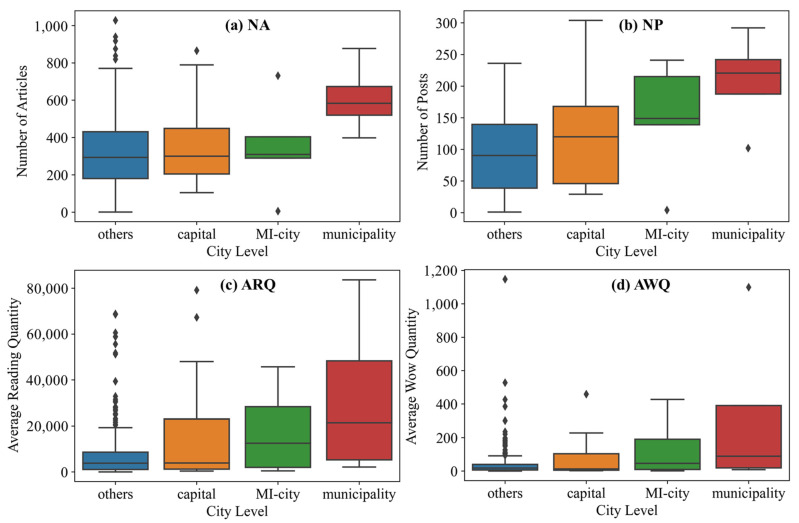
Differences in the emergency response and risk communication in different cities. Note: The comparison of (**a**) NA, (**b**) NP, (**c**) ARQ and (**d**) AWQ at different city levels.

The distribution diagram of the emergency response of the NA (*p* = 0.069) and NP (*p* = 0.012) of the official WeChat accounts in cities of different levels is shown in Figure 2. Due to differences in the city size, administrative level, organizational setup, and management level, cities of different levels will directly affect their emergency response level. A large city means more people, more traffic, and more urban services, which means the greater movement of people in the face of the pandemic, greater exposure risk, and more difficulty in terms of managing the whole city. The city level significantly affects the emergency response of the city to the pandemic situation, and the higher the city level is, the higher the emergency response level is. The risk level in a city is roughly the same as the emergency response level.

### 3.3. Risk Communication Effects

The statistical analysis of the regional risk communication effects from the perspective of the ARQ and AWQ of the articles published in February 2020 are shown in Table 2.

In provincial media, there are no significant regional differences in terms of the risk communication of different types of accounts. As for municipal media, the ARQ (*p* < 0.001) and AWQ (*p* < 0.001) among all of the cities have regional differences (Figure 1c,d), but key cities have no significant differences. Additionally, the differences are also independent of the cities at different levels. In terms of regional distribution, the ARQ and AWQ show a decreasing trend from EC, SC, MC, NC, NE, and WC. The degree of concern to the development of pandemic information in each province is basically the same, and the main regional differences are at the city level. This is consistent with the differences in the emergency response effects. The consistency of “read” and “wow” indicates that the acceptance of risk communication to the COVID-19 by audiences is similar in different places. Therefore, the existing differences in risk communication may be the result of differences in emergency response.

### 3.4. Temporal Trends in Emergency Response and Risk Communication

**Figure 3:**
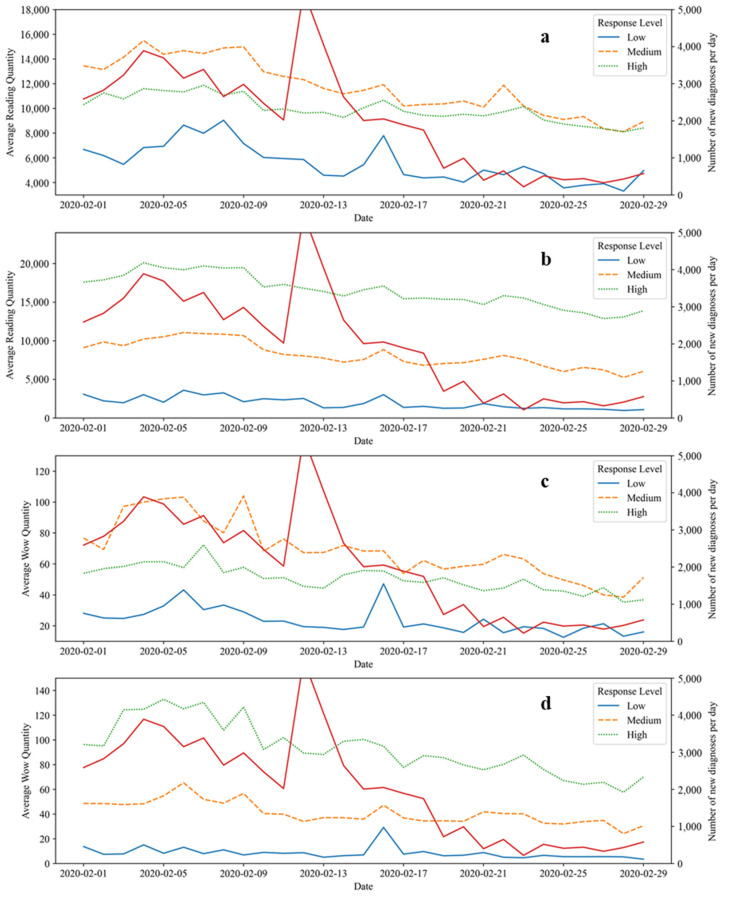
Diurnal variation of risk communication effects under different emergency response levels. Note: The unindicated point in the daily number of new diagnoses of the COVID-19 in China is 15,152 new cases on 12 February 2020. (**a**) The variation of ARQ on the classification of NA. (**b**) The variation of ARQ on the classification of NP. (**c**) The variation of AWQ on the classification of NA. (**d**) The variation of AWQ under time dimension on the classification of NP.

Emergency response, risk communication, and the evolution are all extremely dynamic, so a month total can obscure some important information. In order to more precisely analyze the development and changes in the impact of different emergency response levels on risk communication during the pandemic period, the relevant content in February 2020 was decomposed and analyzed on a daily basis. The diurnal variation of risk communication effects under different emergency response levels is shown in Figure 3.

The public’s attention to the development of the pandemic gradually declined from the perspective of ARQ and AWQ, which means that the effectiveness of risk communication may have deteriorated. However, this does not mean that actual risk communication was less effective. The emergency response of the public sector seeks to communicate the existence, status, and coping strategies for pandemic risks with the general public [11,58]. During the pandemic, there was a constant bombardment of risk communication and information. The Chinese people basically understood its connotation and entered the information reception fatigue period This steady state of declining interest could be a turning point in the pandemic or the prelude greater risk. Fortunately, with China’s strict management measures, China was basically in a state of “zero population flow” in February 2020. The pandemic control pattern in Wuhan was stable and had good momentum, and the number of new diagnoses per day showed a downward trend. The overall pandemic prevention pattern in China was controlled and stable. The essence of numerical reduction effects is the effective accumulation of risk communication and a good state of prevention and control.

Curves based on the NA and NP have obvious grading effects on risk communication (Figure 3). A general conclusion that the more articles the WeChat official accounts post, the higher the corresponding ARQ and AWQ are, is not completely consistent. The ARQ and AWQ of the medium-level group based on NA were higher than those of the high-level group. However, the values of the medium NA group had a faster downward trend, which is roughly in line with that of the high NA group by the end of February (Figure 3a,c). However, the risk communication effects of the group based on NP maintained obvious differences between groups, and risk communication effect was positively correlated with the effect of the public sector’s emergency response level (Figure 3b,d). This shows that the audience of the accounts with a high quantity and low frequency of articles are more likely to enter the information reception fatigue period [35]. Moreover, it also provides effective measures to deal with the information reception fatigue period and improves the effect of risk communication, that is, improves the frequency of communication [59]. The information receiving fatigue period is caused by the public being bombarded with and receiving a large amount and similar information over a period long time, and the corresponding content’s familiarity increases, and interest decreases [60]. Zhang et al. [61] found the negative impact of social fatigue caused by information overload in social media on the effectiveness of audience communication from the perspective of information relevance. Information overload causes information receiving fatigue to occur widely in many social media, not only on WeChat [62,63,64], but also on Facebook [65,66], Twitter [67], etc. Studies have confirmed that there was an information reception fatigue period that was caused by the overload of social media information during the COVID-19 that resulted in the effect of risk communication being reduced and adversely affected the prevention and control of the pandemic [68,69,70]. By increasing the frequency of risk communication and releasing relevant content immediately after the occurrence of each event, the public sector can capture public interest with higher information quality, relieve the fatigue of public information reception, and improve the effect of risk communication [70,71,72].

### 3.5. Risk Communication Effects of Different Types of Articles

**Figure 4:**
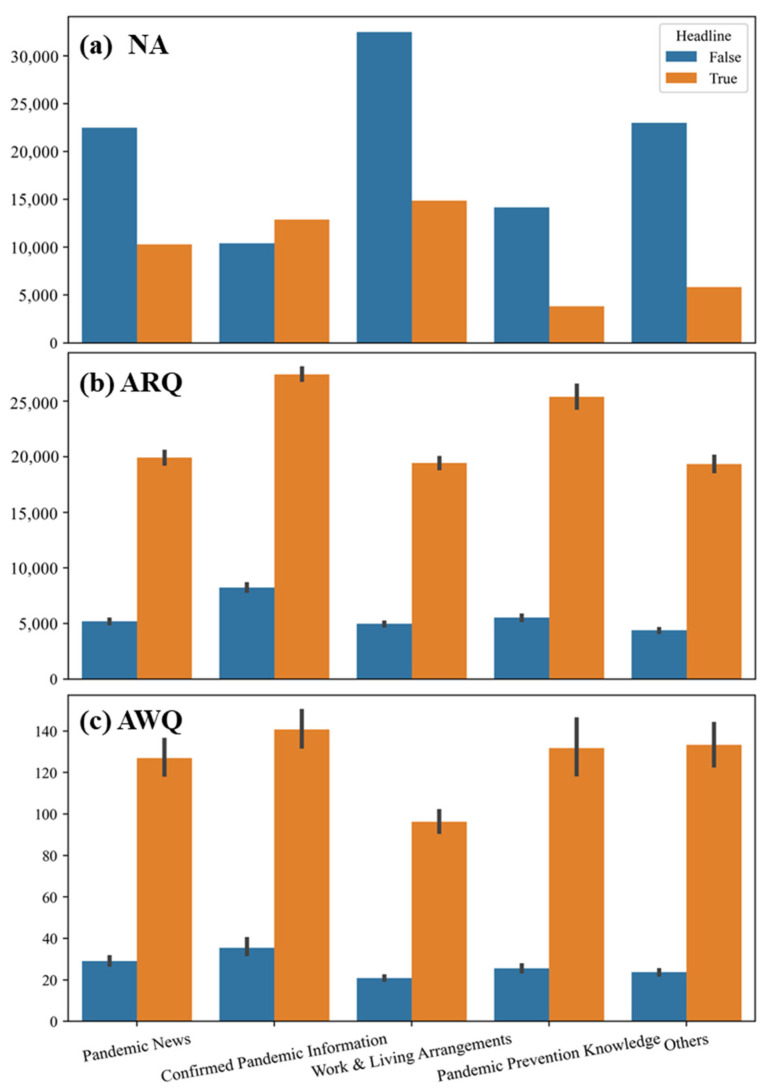
Effects of different types of articles on emergency response and risk communication. Note: The comparison of (**a**) NA, (**b**) ARQ and (**c**) AWQ at different types of articles.

Different article types were significant for both ARQ (H = 7372.501, *p* < 0.001) and AWQ (H = 4980.387, *p* < 0.001), indicating the impact of article types on risk communication during the pandemic (Figure 4). In terms of NA, the headlines mainly focused on pandemic news, confirmed pandemic information, and work and living arrangements. The number of headlined articles with confirmed pandemic information even exceeded the number of non-headlined articles. Moreover, it also obtained the highest ARQ and AWQ, indicating the importance of transparent and timely pandemic data for risk communication during the outbreak period in China in February 2020. For the content related to pandemic prevention knowledge, although the number of headlined articles was small, still had a high risk communication effect, which met the public’s demand for relevant knowledge during the pandemic. Despite a high number of non-headlined articles about work and living arrangements, they did not achieve a good communication effect. There were two main possible reasons for this. The first one was that the specific arrangements involved in these articles were related to industries and jobs. Additionally, the second reason for this could be that for China, which is under embargo, most audiences paid less attention to the subsequent arrangements. For pandemic news, the main difference was whether the article had a headline or not. Usually, headlines indicate the urgency and the importance of the news and will naturally attract higher attention than non-headlined articles. In general, the adoption of different types of articles by the public is different. The public pays more attention to timely news, confirmed pandemic information, and health management suggestions for their own health, which also indicates a direction for us to further improve the effect of risk communication and to help control the pandemic.

### 3.6. Factors Affecting Emergency Response and Risk Communication

GDP, resident population, per capita GDP (pGDP), education level, literacy level, cumulative number of diagnosed, and newly diagnosed cases in February 2020 were selected as influencing factors to investigate a city’s emergency response and risk communication [6,11,19,73]. The cumulative number of diagnosed patients was analyzed for February 1, February 15, and March 1, respectively. The statistical analysis of the impact of each influencing factor on emergency response is shown in Table 3, and Table 4 lists the statistical analysis results of the impact of each influencing factor on risk communication.

With the exception of resident population (*p* = 0.081) and pGDP (*p* = 0.629), the other factors had significant effects in terms of emergency response. All of these factors had a significant impact on risk communication. Usually, GDP represents the volume of the economy, while pGDP represents the intensity of the economy. Economic intensity affected the number of posts but did not significantly affect the number of articles. This may mean that when the economy is sufficient, the task of quantity is first completed in the pandemic emergency response, while the response frequency to better reflect the role of the emergency response needs the support of economic intensity. In terms of pandemic control, relying on a high-frequency emergency response to improve the effect of risk communication is a requirement of fine urban management, which requires the improvement of the comprehensive strength of cities represented by pGDP. City size affects city management difficult and the risk of infection. Education level reflects people’s awareness of the pandemic, their ability to implement government policies, and their ability to judge their own protection. The construction of local media platforms such as WeChat public platforms, the active situation of media, and the mode of policy publicity are influenced by educational level. The local number of diagnosed cases reflects the source of inflection, the risk of infection and the urgency of pandemic prevention, and control of COVID-19 in cities. Moreover, it also influences people’s understanding of the urgency of pandemic. These factors affect the effect of emergency response and risk communication from multiple perspectives, such as the background, foundation, communication process, and audience status. Making good use of the influence of these factors is an important measure for government decision-makers to design risk communication strategies to improve the effectiveness of non-pharmaceutical interventions in the prevention and control of the pandemic [74].

### 3.7. Evaluation of Emergency Response and Risk Communication Effects

#### 3.7.1. Reading Conversion Rate

The reading conversion rate means that readers rank the posts “wow” after they receive and understand the post information to ensure that the posts are more widely spread. It reflects the degree to which reader agree with the content of the articles. The reading conversion rate shows significant regional differences (H = 18.598, *p* = 0.002), but it is not related to city-level differences (H = 1.626, *p* = 0.653). It is consistent with previous conclusions that regional differences in terms of risk communication are greater than they are in terms of emergency response (Figure 1), which leads to differences in the reading conversion rate. Differences in terms of pandemic status, education, and culture are all among important reasons for changes in the reading conversion rate.

#### 3.7.2. Headlined Posts Differences

WeChat official accounts can post several articles at one time. When posted on WeChat platform, headlined articles are displayed with a title, a large photo and a brief introduction while the non-headlined articles have only one title with a small photo. The differences in display form make people pay more attention to headlines when they quickly browse the application. Then, readers enter the profiles official accounts to read the content. In terms of pandemic risk communication, the median ARQ of headlined articles in February 2020 was 4.4 times greater than that of non-headlined articles (Z = −15.490, *p* < 0.001), and the AWQ was 4.0 times greater (Z = −15.385, *p* < 0.001). Although the reading conversion rate was lower than that of non-headlined articles (Z = −3.069, *p* = 0.002), there is no doubt about the advantages of the dissemination effects of the headlined posts. In terms of emergency response, a good grasp of the advantages of headlined articles is conductive to improving the effectiveness of risk communication. For example, the timely posting of news such as pandemic data in the headlines or health knowledge articles that do not have very attractive headlines using the attraction of the headlines for drainage. The readers who are interested in this content could choose to read it in this way, so the reading conversion rate will also rise.

### 3.8. The Role of the Government and Public in Emergency Response and Risk Communication

#### 3.8.1. Government Leading

Chinese governments at all levels are the policy makers and implementors of the prevention and control of the pandemic, which are their functions [75,76]. The government is the information source for news, data statistics, and measures that have been implemented for pandemic control and is the agent of emergency response and the initiator of risk communication [77]. Different types of media are the integrators of medium and information. The fragmentation of functions at all levels of government for efficient operation results in overlapping information releases. Both the work of national government departments that cover the provincial level and the media that report on the provincial level units lead to the decline of the emergency response effect of the media in the province. The same is true for provincial cities, whose emergency response functions are overridden by superiors because the information has already been released by provincial authorities. On the other hand, the outbreak of the COVID-19 pandemic has boosted the emergency response capacity across China. A number of cities without government accounts have set up their own WeChat official accounts [77]. The content and form of emergency response release have also been standardized gradually.

#### 3.8.2. Audience Assessment

Everyone needs to make efforts to bring the pandemic under control. The public is the recipient of emergency response and risk communication, and they also pay close attention to the development of the crisis [78]. During the pandemic, great attention should be paid to the response of the public [79]. The media should give audiences correct answers when they are in a pandemic confusion period [80,81]. During periods of information reception fatigue, they need to explore the concerns of the masses for information drainage. Information publishers should always consider the needs of readers’ lives and ease their anxiety [58,71,82]. Increasing public acceptance through various means is important in the fight against the pandemic [83]. One of the most effective ways to effectively control the pandemic in Wuhan, where the pandemic first broke out, promotes public participation through risk communication and facing the uncertain pandemic situation together [84].

## 4. Conclusions

This is the first study investigating the spatial distribution of the effectiveness of emergency response and risk communication via social media in China in February 2020, when the existing COVID-19 infected cases peaked in China. With data generated from official accounts on the biggest social media platform, WeChat, we found that the cities in East China and the other more developed cities performed better regarding emergency response and risk communication. Such regional differences were positively correlated with the local economy, education, and culture levels as well as with the potential exposure risk to the pandemic in a given region. For different types of accounts, articles pushed by government accounts were the most influential among the public followed by those published by health commissions and news media accounts. It is worth mentioning that the over-bombardment of information may lead to information receiving fatigue for the public. Improving the timeliness of news and the usage of more attractive headlined articles are warranted for more effective emergency response and risk communication regarding the COVID-19 management.

## 5. Limitations and Further Research

Limits also exist. Although attention has been paid to audience coverage in terms of accounts screening, it may not be representative of the overall situation of the local area. The impact of the density of the urban population on the attention paid to WeChat official accounts and the corresponding article data is worth paying attention to, although the media that provide services will play a role in diversion as the urban population increases. In addition, WeChat is among the most influential social media in China, but some of the selected WeChat official accounts may not be subscribed to by that many users. Therefore, more social media platforms and accounts can be selected for future research, not only on WeChat, but also on Weibo, Facebook, Twitter, etc. A larger range of WeChat official accounts, including those from nongovernmental individuals and/or organizations, could also be considered. On the one hand, they reflect the information that the audience urgently needs. On the other hand, we can also pay attention to the negative impact of the spread of the misinformation they release.

## Figures and Tables

**Table 1:** Statistical analysis of emergency response effects.

	Number of Articles		Number of Posts	
	Test Statistic	*p* Value	Test Statistic	*p* Value
(a) Provincial government accounts	1.827	0.873	2.602	0.761
(b) Provincial news media accounts	13.769	0.017	10.876	0.054
(c) Provincial health commission accounts	4.035	0.544	10.425	0.064
(d) Accounts from all cities	12.600	0.027	38.450	<0.001
(e) Government accounts in key cities	3.032	0.695	6.142	0.293
(f) News media accounts in key cities	3.010	0.698	3.895	0.565
(g) Accounts from all cities at different levels	7.107	0.069	10.905	0.012

**Table 2:** Statistical analysis of risk communication effects.

	Average Reading Quantity		Average Wow Quantity	
	Test Statistic	*p* Value	Test Statistic	*p* Value
(a) Provincial government accounts	3.847	0.572	4.267	0.512
(b) Provincial news media accounts	9.647	0.086	4.659	0.459
(c) Provincial health commission accounts	6.657	0.247	6.508	0.260
(d) Accounts from all cities	33.989	<0.001	22.141	<0.001
(e) Government accounts in key cities	6.066	0.300	4.880	0.431
(f) News media accounts in key cities	2.121	0.832	1.813	0.874
(g) Accounts from all cities at different levels	5.815	0.121	3.815	0.282

**Table 3:** Statistical analysis of the influencing factors of emergency response effects.

	Number of Articles		Number of Posts	
	Test Statistic	*p* Value	Test Statistic	*p* Value
GDP	10.029	0.040	39.765	<0.001
Resident population	8.319	0.081	29.551	<0.001
pGDP	2.586	0.629	26.954	<0.001
Education level	17.256	0.002	52.470	<0.001
Culture level	11.421	0.022	62.402	<0.001
Accumulated diagnosis on 1 February 2020	15.236	0.004	53.357	<0.001
Accumulated diagnosis on 15 February 2020	23.744	<0.001	58.600	<0.001
Accumulated diagnosis on 1 May 2020	20.132	<0.001	54.865	<0.001
Diagnosed on February 2020	14.733	0.005	50.627	<0.001

**Table 4:** Statistical analysis of the influencing factors of risk communication effects.

	Average Reading Quantity		Average Wow Quantity	
	Test Statistic	*p* Value	Test Statistic	*p* Value
GDP	48.954	<0.001	38.357	<0.001
Resident population	41.248	<0.001	26.877	<0.001
pGDP	28.662	<0.001	24.130	<0.001
Education level	52.807	<0.001	42.416	<0.001
Culture level	55.472	<0.001	47.172	<0.001
Accumulated diagnosis on 1 February 2020	58.829	<0.001	40.811	<0.001
Accumulated diagnosis on 15 February 2020	65.413	<0.001	48.104	<0.001
Accumulated diagnosis on 1 May 2020	64.643	<0.001	46.999	<0.001
Diagnosed on February 2020	63.780	<0.001	46.474	<0.001

## Data Availability

The relevant data from this study come from the public data collation of the WeChat public platform.
